# Oligo-Fucoidan supplementation enhances the effect of Olaparib on preventing metastasis and recurrence of triple-negative breast cancer in mice

**DOI:** 10.1186/s12929-022-00855-6

**Published:** 2022-09-15

**Authors:** Li-Mei Chen, Pao-Pao Yang, Aushia Tanzih Al Haq, Pai-An Hwang, You-Chen Lai, Yueh-Shan Weng, Michelle Audrey Chen, Hsin-Ling Hsu

**Affiliations:** 1 Institute of Molecular and Genomic Medicine, National Health Research Institutes, 35 Keyan Road, Miaoli County, Zhunan, 35053 Taiwan; 2 Department of Bioscience and Biotechnology, National Taiwan Ocean University, Keelung, Taiwan

**Keywords:** Oligo-Fucoidan, Olaparib, Triple-negative breast cancer, Cancer stem cells, M1/M2 macrophage polarization, Glucose uptake, Lactate production, IL-6/EGFR/PD-L1 signaling pathway

## Abstract

**Background:**

Seaweed polysaccharides have been recommended as anticancer supplements and for boosting human health; however, their benefits in the treatment of triple-negative breast cancers (TNBCs) and improving immune surveillance remain unclear. Olaparib is a first-in-class poly (ADP-ribose) polymerase inhibitor. Oligo-Fucoidan, a low-molecular-weight sulfated polysaccharide purified from brown seaweed (*Laminaria japonica*), exhibits significant bioactivities that may aid in disease management.

**Methods:**

Macrophage polarity, clonogenic assays, cancer stemness properties, cancer cell trajectory, glucose metabolism, the TNBC 4T1 cells and a 4T1 syngeneic mouse model were used to inspect the therapeutic effects of olaparib and Oligo-Fucoidan supplementation on TNBC aggressiveness and microenvironment.

**Results:**

Olaparib treatment increased sub-G1 cell death and G2/M arrest in TNBC cells, and these effects were enhanced when Oligo-Fucoidan was added to treat the TNBC cells. The levels of Rad51 and programmed death-ligand 1 (PD-L1) and the activation of epidermal growth factor receptor (EGFR) and adenosine 5′-monophosphate (AMP)-activated protein kinase (AMPK) facilitate drug resistance and TNBC metastasis. However, the combination of olaparib and Oligo-Fucoidan synergistically reduced Rad51 and PD-L1 levels, as well as the activity of EGFR and AMPK; consistently, TNBC cytotoxicity and stemness were inhibited. Oligo-Fucoidan plus olaparib better inhibited the formation of TNBC stem cell mammospheroids with decreased subpopulations of CD44^high^/CD24^low^ and EpCAM^high^ cells than monotherapy. Importantly, Oligo-Fucoidan plus olaparib repressed the oncogenic interleukin-6 (IL-6)/p-EGFR/PD-L1 pathway, glucose uptake and lactate production. Oligo-Fucoidan induced immunoactive and antitumoral M1 macrophages and attenuated the side effects of olaparib, such as the promotion on immunosuppressive and protumoral M2 macrophages. Furthermore, olaparib plus Oligo-Fucoidan dramatically suppressed M2 macrophage invasiveness and repolarized M2 to the M0-like (F4/80^high^) and M1-like (CD80^high^ and CD86^high^) phenotypes. In addition, olaparib- and Oligo-Fucoidan-pretreated TNBC cells resulted in the polarization of M0 macrophages into CD80(+) M1 but not CD163(+) M2 macrophages. Importantly, olaparib supplemented with oral administration of Oligo-Fucoidan in mice inhibited postsurgical TNBC recurrence and metastasis with increased cytotoxic T cells in the lymphatic system and decreased regulatory T cells and M2 macrophages in tumors.

**Conclusion:**

Olaparib supplemented with natural compound Oligo-Fucoidan is a novel therapeutic strategy for reprogramming cancer stemness, metabolism and the microenvironment to prevent local postsurgical recurrence and distant metastasis. The combination therapy may advance therapeutic efficacy that prevent metastasis, chemoresistance and mortality in TNBC patients.

**Supplementary Information:**

The online version contains supplementary material available at 10.1186/s12929-022-00855-6.

## Background

Triple-negative breast cancers (TNBCs) are clinically negative for the expression of estrogen receptor, progesterone receptor and human epidermal growth factor receptor 2 [1]. This type of cancer lacks proper therapeutic targets and shows the worst overall survival of all breast cancers. Currently, metastatic TNBC treatment is limited to surgery, chemotherapy and radiation, which has no significant or major therapeutic benefits for patients, and treated patients still face chemoresistance and high rates of relapse, metastasis and mortality. Few systemic treatment options except chemotherapy have been approved for targeting TNBCs. Therefore, identifying systematic therapeutic regimes, supplements and novel targets to enhance clinical use is urgently needed. TNBCs are associated with hyperactive EGFR and MEK/ERK and sensitivity to DNA-damaging agents [2]. Thus, targeting the DNA repair process and EGFR/MEK/ERK signaling pathway are recommended to treat TNBCs. Ongoing clinical trials for TNBCs have targeted androgen receptor, EGFR, PARP, FGFR and the angiogenic pathways [1]. Poly (ADP-ribose) polymerase (PARP) inhibitors have been recognized as the most promising cures for BRCA-associated cancers and sporadic TNBCs [3, 4]. Olaparib is the most well-investigated PARP inhibitor in the treatment of metastatic BRCA-mutated breast cancers and TNBCs [5]. As olaparib inhibits PARP enzymes, it prevents DNA damage repair and causes the cancer cells to die.

The inflammatory microenvironment impacts cancer progression and metastasis. TNBCs are very immunogenic and have abundant tumor-infiltrating lymphocytes, which affect clinical outcome and patient survival [6]; thus, immunotherapy is also a promising strategy for TNBCs [7]. Programmed death-ligand 1 (PD-L1) is abundant in TNBCs that maintains the immunosuppressive tumor microenvironment (TME) [8]. Targeting the glycosylated PD-L1 protein by a monoclonal antibody (Ab) enhances the immune checkpoint in TNBC cells that blocks the PD-L1/PD-1 (programmed death-1) interaction and promotes the internalization and degradation of PD-L1. A phase 2 trial (NCT03167619) examined the efficacy of olaparib combined with durvalumab (anti-PD-L1) in the treatment of advanced TNBCs, and the results indicated that anti-PD-L1 and PARP inhibitor together increase antitumor immunity. Moreover, metformin (an activator of AMP-activated protein kinase (AMPK)) reversed epithelial-mesenchymal transition (EMT) and PD-L1 induction in PARP inhibitor-resistant TNBC cells by inhibiting the phosphorylation on Serine 473 residue of AKT (Ser473), which sensitized PARP inhibitor-resistant cells to cytotoxic T cells [4].

Macrophages are the major immunoregulatory innate immune cells in TME. Tumor-associated macrophages (TAMs) are derived from circulating monocytes that are recruited and accumulate in tumors [9, 10], where they promote tumor progression, angiogenesis, tissue remodeling, immune evasion, drug refractoriness and metastasis. Depending on the microenvironmental factors, macrophages are polarized into M1 (classically activated) or M2 (alternatively activated) phenotype. Infiltrating immunosuppressive M2 macrophages in the TME promote cancer progression, whereas inflammatory M1 macrophages are tumoricidal. The ratio of M1/M2 TAMs in human cancer could determine clinical outcomes; thus, the application of macrophage-based therapeutic strategies is important for effective cancer treatment.

Metabolic reprogramming in cancer cells and stromal cells is required for adapting to energy expenditure and malnutrition during cancer progression [11]. Enhanced glucose uptake accelerates cancer cell proliferation, invasion and migration [11]. To meet high energy demands, glucose metabolism in cancer cells works through aerobic glycolysis or the Warburg effect in the cytoplasm rather than depending on mitochondrial oxidative phosphorylation (OXPHOS), as do normal cells [12]. Glucose transporter 1 (GLUT1) is overexpressed in aggressive basal-like TNBC, mediates glucose passage across the plasma membrane and promotes TNBC cell expansion and malignancies [13–15]. Activation of EGFR and AKT promotes aerobic glycolysis by stabilizing GLUT1 in the cell membrane and inhibiting GLUT1 endocytosis [16]. After glucose is imported into cancer cells, it undergoes glycolysis to produce pyruvate that is primarily diverted to lactate fermentation in the cytoplasm rather than to OXPHOS in mitochondria, facilitating the rapid generation of ATP and sufficient glycolytic intermediates to support anabolic demands of cancer cells [17].

Fucoidans are a class of abundant polysaccharides in brown seaweed that have been shown to have anti-inflammatory, antitumor, antiviral, anticoagulant, antithrombotic, antiobesity and antiangiogenic functions [18–24]. As immune modulators, fucoidans may activate natural killer cells, macrophages and cytotoxic T cells [25], which will enhance chemotherapeutics against tumor growth. By suppressing the NF-κB/CCL22 axis in M2 macrophages [25], the fucoidan limits cancer cell mobility and reduces intratumoral CD4(+) regulatory T cells (Tregs) to enhance cancer therapeutics. While BALB/c mice are treated with the fucoidan, immune cell expansion, IL-2 secretion, macrophage phagocytosis and serum antibodies (Abs) (IgM, -G, -A) are increased [26].

Oligo-Fucoidan (low-molecular-weight fucoidan or LMF) is a sulfated fucose-rich polysaccharide that is isolated from the brown seaweed *Sargassum hemiphyllum* [27, 28] or *Laminaria japonica* [29, 30]. Oligo-Fucoidan purified from *Sargassum hemiphyllum* is not toxic to normal hepatocytes L02 cell viability [31]. Also, Oligo-Fucoidan purified from *Laminaria japonica* has not toxicity in NIH3T3 cells [29] and no harmful effect on the host defense system that spleen-to-body-weight ratio remains constant in the treated mice [30]. We have found that Oligo-Fucoidan coordinates with p53 to further prevent tumor progression in immunodeficient mice bearing human colon HCT116 cancer cells [27, 28], and Oligo-Fucoidan exerts antioxidant, anti-inflammatory and antiangiogenic effects [28]. We also identified that Oligo-Fucoidan supplementation reduced the adverse effect of chemotherapy, which suppressed monocyte chemoattractant protein-1 (MCP-1/CCL2) and the proinflammatory cytokine IL-6 expression [27]. Importantly, Oligo-Fucoidan combined with the chemotherapy restricts intratumoral M2 macrophages in xenograft mice [27, 28], and supports immune surveillance by impairing M2 polarization and invasiveness and repolarizes macrophages toward the M1-like phenotype in vitro [28]. Thus, Oligo-Fucoidan is an ideal immunomodulator that will restore a healthy microenvironment, activate tumor suppressor and reprogram cytokine profile, which benefits antitumor immunity.

Here, we first identified an innovative strategy using the natural supplement Oligo-Fucoidan plus a PARP inhibitor (olaparib) for controlling TNBC growth, metastasis and recurrence. Oligo-Fucoidan and olaparib together impaired cancer stemness, glucose metabolism and the oncogenic pathway better than monotherapy. Combining Oligo-Fucoidan with chemotherapy and/or immunotherapy may improve clinical outcomes by increasing immune checkpoint and therapeutic efficacy.

## Methods

### Cell lines and culture condition 

Human TNBC cell lines MDA-MB-231 (ATCC, Manassas, VI, USA) and HCC1395 (ATCC), murine TNBC cell line 4T1 (ATCC) and human THP-1 monocytes (ATCC) were maintained in RPMI-1640 medium (Life Technologies, Grand Island, NY, USA) with 10% fetal bovine serum (FBS) (Life Technologies), 2 mM l-glutamine (HyClone, South Logan, UT, USA), 100 units/ml penicillin (HyClone) and 100 μg/ml streptomycin (HyClone) in a 37 °C incubator with 5% CO_2_.

### Primary antibodies for Western blotting

Ab against p21 was purchased from Santa Cruz Biotechnology (Dallas, Texas, USA). Antibodies (Abs) against Arginase-1, cleaved PARP (Asp214), EGFR, iNOS, IL-10, PARP, p38 MAPK, phospho-AMPK (Thr172), phospho-EGFR (Tyr1068), PD-L1, phospho-Histone H2AX (Ser139) (γ-H2AX), phospho-p38 MAPK (Thr180/Tyr182), phospho-PKM2 (Tyr105), PKM2 and Rad51 were obtained from Cell Signaling (Danvers, MA, USA). Abs against β-actin, AMPK and IL-6 were purchased from GeneTex, Inc. (Irvine, CA, USA). Abs against GluT1, HK2, PFKL, RPIA and MCT4 were obtained from Elabscience (Houston, TX, USA). All the above Abs were used for Western blotting. SDS–PAGE and immunoblotting were conducted as described previously [32].

### Oligo-Fucoidan preparation

Oligo-Fucoidan (also known as Low-Molecular-Weight Fucoidan LMF) was purified from *Laminaria japonica* seaweed sample using similar approaches as previously described [29, 30]. Briefly, 100 g of dried seaweed power were suspended in 5 liter of distilled water and boiled at 100 °C for 30 min, centrifuged at 10,000×*g* for 20 min, and the supernatant was incubated with 4 M CaCl_2_ for 1 h to separate alginic acid by centrifugation at 10,000×*g* for 20 min. All the supernatants were dialyzed in deionized water for 48 h in a molecular weight (MW) cut-off of 10 kDa dialysis tube. The crude Fucoidan extracts were precipitated by ethanol at a ratio of 1:3 (V/V) and then fractionated through anion‐exchange chromatography using DEAE‐Sephadex A‐25 (Sigma–Aldrich) equilibrated with 20 mM Tris–HCl, pH8.0 and eluted with 1.5 M NaCl as described by Hwang et al. [30], and the fraction 3 of eluates were hydrolyzed with the crude glycolytic enzyme isolated from *Bacillus subtilis* [30], into Oligo-Fucoidan with an average MW of 1.2 kDa (~ 90.1%). Oligo-Fucoidan was composed of sulfate (35.4% ± 1.3% (w/w)) and neutral monosaccharides, including fucose (38.71 ± 0.41%), glucose (6.09 ± 0.52%), galactose (19.19 ± 0.31%), myo-inositol (2.95 ± 0.54%), mannose (28.78 ± 0.71%), xylose (3.46 ± 0.34%) and rhamnose (0.82 ± 0.30%). The ration of monosaccharide contents were calculated from the following equation: mole of monosaccharide/mole of (fucose + glucose + galactose + myo-inositol + mannose + xylose + rhamnose) × 100%. The purified Oligo-Fucoidan was dissolved in phosphate-buffered saline (PBS), stirred for 30 min at room temperature and sterilized using filtration before performing experiments.

### Mammosphere formation

4T1 and MDA-MB-231 cells (5 × 10^4^) were cultured in 6-well ultralow attachment plates (Corning) (Corning, NY, USA) in serum-free DMEM/F12 medium (Thermo Fisher Scientific, Waltham, MI, USA) supplemented with 1% l-glutamine, 1% penicillin/streptomycin, 2% B27 (Invitrogen, Carlsbad, CA, USA), 20 ng/ml EGF (Sigma–Aldrich, St. Louis, MO, USA) and 20 ng/ml FGFb (PeproTech, Rehovot, Israel) and treated with 50 μM olaparib (Selleckchem, Houston, TX, USA) and/or 400 μg/ml Oligo-Fucoidan for 5 or 16 days, respectively. The number of mammospheres (≥ 50 μm) was counted using ImageJ software (http://rsb.info.nih.gov/ij/index.html). Photographs were taken with a Nikon DIAPHOT300 inverted microscope at magnifications of 100 × (for 4T1 cells) and 200 × (for MDA-MB-231 cells). All experiments were performed in triplicate, and mammospheres were quantified in 8–10 randomly selected fields.

Mammosphere cells (1 × 10^5^) were harvested and further stained with Alexa Fluor^®^ 647-conjugated anti-human CD24 Ab (BD Pharmingen, San Jose, CA, USA), FITC-conjugated anti-human CD44 Ab (BD Pharmingen) or BB515-conjugated anti-human CD326 Ab (EpCAM) (BD Pharmingen) for 1 h on ice, rinsed with PBS, resuspended in 500 μl of PBS and analyzed by the BD FACSCalibur flow cytometer (BD Biosciences, San Jose, CA, USA). CD44-FITC and CD326-BB515 were excited at 488 nm, and the emissions were measured by FL1 PMT (515–545 nm bandpass filter). CD24-Alexa 647 was excited at 633 nm, and the emission was measured by FL-4 PMT (653–669 nm bandpass filter). CellQuest software (BD Biosciences) and FlowJo software (BD Biosciences) were used to determine the subpopulations of CD44(+)/CD24(−) and EpCAM(+) cells, respectively.

### Colony assay of cell viability

TNBC cells were plated on 6-well plates (5 × 10^3^ cells) and treated 50 μM olaparib and/or 400 μg/ml Oligo-Fucoidan for 14 days. Cell colonies were fixed with 4% paraformaldehyde in PBS for 30 min at 4 °C, washed three time with PBS, stained with 0.2% crystal violet for 2 h, photographed with an optical microscope and quantified by ImageJ software.

### WST-1 cell viability assay

MDA-MB-231 cells were treated with olaparib (0 ~ 50 μM) or 50 μM olaparib and/or 400 μg/ml Oligo-Fucoidan for 24 h and incubated with WST-1 (4[3-(4-iodophenyl)-2(4-nitrophenyl)-2*H*-5-tetrazolio]-1,3-benzene disulfonate) assay solution (Sigma–Aldrich) for 1 h. The tetrazolium salt was cleaved to form formazan by cellular mitochondrial dehydrogenase, the formazan dye was quantitated with a scanning multiwell spectrophotometer. The measured absorbance directly correlates to the number of viable cells.

### Cell cycle analysis

MDA-MB-231 cells were treated with 50 μM olaparib and/or 400 μg/ml Oligo-Fucoidan for 48 h. The cells were fixed with 70% ethanol at − 20 °C for 1 h and resuspended in PBS containing 0.1% (v/v) Triton X-100 (Sigma–Aldrich), 5 μg/ml DNase-free RNase A (Sigma–Aldrich) and 10 μg/ml propidium iodide (PI) (Thermo Fisher Scientific) in the dark for 30 min. PI fluorescence was excited at 543 nm, and the emissions at 615 nm were measured by the FL2 PMT channel and quantified with the BD FACSCalibur flow cytometer.

### Quantitative real-time polymerase chain reaction (qRT–PCR)

Total RNA was extracted using TRIzol™ reagent (Invitrogen). The RNA samples were quantified using a NanoDrop ND1000 spectrophotometer (Thermo Fisher Scientific). An aliquot of total RNA (1 μg) was reversely transcribed using a Maxima First Strand Synthesis kit (Thermo Fisher Scientific) at 25 °C for 10 min, 50 °C for 15 min and 85 °C for 5 min before chill on ice for 10 min. qRT–PCR was conducted using SYBR Green Master Mix (Roche Diagnostics, Basel, Switzerland) and analyzed by an ABI ViiA Thermal Cycler (Applied Biosystems, Foster City, CA, USA). qRT–PCR was performed at 95 °C for 15 min, followed by 40 cycles at 95 °C for 15 s and 60 °C for 1 min. β-actin level was used as an internal control. Relative mRNA level was calculated by the following formula: ΔΔCT = ΔCt test sample-ΔCt control sample. Fold changes in gene expression were calculated using the comparative 2^−ΔΔCT^ method.

The primers were synthesized by PURIGO Biotechnology (Taipei, Taiwan). The primer sequences of amplicons for human ALDH1A1 (forward: 5′-CACGCCAGACCTACCTGTCC-3′; reverse: 5′-GCAGAGCTCCTCAGTTG-3′), CD44 (forward: 5′-TTACAGCCTCAGCAGAGCAC-3′; reverse: 5′-TGACCTAAGACGGAGGGAGG-3′), CD24 (forward: 5′-CTCACAGAACAAAGCAAGGGC-3′; reverse: 5′-GCCTAGCGCGAACCCTCC-3′), EpCAM (forward: 5′-TGCTGGAATTGTTGTGCTGG-3′; reverse: 5′-AGATGTCTTCGTCCCACG-3′), Nanog (forward: 5′-TGGGAAGAAGCTAAAGAGCCAG-3′; reverse: 5′-GGATGCTTCAAAGCAAGGCA-3′), Sox2 (forward: 5′-CATGAAGGAGCACCCGGATT-3′; reverse: 5′-TTAATGTGCGCGTAACTGTG-3′), Snail (forward: 5′-GCGAGCTGCAGGACTCTAAT-3′; reverse: 5′-GGACAGAGTCCCAGATGAGC-3′), F4/80 (forward: 5′-CAATGAGTGCCTCACCAGCA-3′; reverse: 5′-TGGGCAAGCTCTTGGATCTG-3′), CD80 (forward: 5′-GCAGGGAACATCACCATCCA-3′; reverse: 5′-TCACGTGGATAACACCTGAACA-3′), CD86 (forward: 5′-GCTTTGCTTCTCTGCTGCTG-3′; reverse: 5′-GGCAGGTCTGCAGTCTCATT-3′), CD163 (forward: 5′-CCGGGAGATGAATTCTTGCCT-3′; reverse: 5′-AGACACAGAAATTAGTTCAGCAGCA-3′), CD206 (forward: 5′-CTGAATTGTACTGGTCTGTCCT-3′; reverse: 5′-GCTTAGATGTGGTGCTGTGG-3′), TGF-β (forward: 5′-TTGACTTCCGCAAGGACCTC-3′; reverse: 5′-CTCCAAATGTAGGGGCAGGG-3′) and β-actin (forward: 5′-CACCAGGGCGTGATGGTGGG-3′; reverse: 5′-GATGCCTCTCTTGCTCTGG GC-3′) were designed according to the NCBI Probe database.

### Analysis of the macrophage polarity

Human THP-1 monocytes (1 × 10^5^) were treated with 50 μM olaparib and/or 400 μg/ml Oligo-Fucoidan for 48 h. Polarized macrophages were stained with FITC-conjugated anti-CD80 Ab and Alexa Fluor 647-conjugated anti-CD163 Ab (BD Pharmingen) were diluted (1:50) in staining buffer (1% FBS in PBS) for 1 h on ice. Fluorescence intensity was quantified by the BD FACSCalibur flow cytometer in the FL1 and FL4 channels at excitation/emission wavelengths of 488 nm and 633 nm, respectively. The results were quantified using FlowJo software.

THP-1 monocytes were treated with 100 ng/ml phorbol myristate acetate (PMA) (GeneTex, Inc.) for 72 h to induce M0 macrophage differentiation. M0 macrophages were stained with Alexa Fluor 546-conjugated anti-F4/80 Ab (BD Pharmingen) excited by a 488-nm laser line, and the emissions were measured in the FL2 channel (564–606 nm) with a 585/42 BP filter by the BD FACSCalibur flow cytometer.

MDA-MB-231 cells (4 × 10^5^) were pretreated with 50 μM olaparib and/or 400 μg/ml Oligo-Fucoidan for 48 h, the treatments were rinsed off, conditioned medium (CM) was collected, and the CM was incubated with M0 macrophages for 48 h. The phenotypes of CD80(+) M1 and CD163(+) M2 macrophages were measured by flow cytometry as described above.

### Trajectories of cancer cell migration

MDA-MB-231 cells (1 × 10^5^) were treated with 50 μM olaparib and/or 400 μg/ml Oligo-Fucoidan in RPMI medium containing 0.5% serum for 48 h. Cell mobility was analyzed with a Leica AF6000LX microscope (Leica Microsystems, Wetzlar, Germany) using a 20 × objective. Images were acquired for 18–24 h at 10-min intervals. Cell movements were tracked with Metamorph (Molecular Devices, San Jose, CA, USA) to quantify the total migration distances. Cell trajectories emanating from the initial position were plotted using the DiPer macro [33].

### Glucose uptake assay

A glucose uptake cell-based assay kit (Cayman Chemical, Ann Arbor, MI, USA) was used to analyze cellular glucose uptake. Briefly, cells (5 × 10^4^/well) were seeded in 96-well plates and incubated overnight in 100 μl of glucose-free medium, reacted with 200 μg/ml 2-NBDG (2-deoxy-2-[(7-nitro-2,1,3-benzoxadiazol-4-yl)amino]-d-glucose) for 1 h in glucose-free medium and then centrifuged for 5 min at 400×*g* at room temperature to remove the supernatant. Two hundred microliters of the assay buffer was added to each well, the plate was centrifuged for 5 min at 400×*g* at room temperature, the supernatant was aspirated, and 100 μl of assay buffer was added to each well. 2-NBDG fluorescence was quantified using the infinite M200 PRO (TECAN, Männedorf, Switzerland) with excitation/emission wavelengths of 485/525 nm.

### Lactate production assay

An l-Lactic acid (LA) colorimetric assay kit (ACE biolabs, Taoyuan, Taiwan) was used to measure LA levels. Briefly, 4T1 or MDA-MB-231 cells (4 × 10^5^) were cultured in medium containing 10% FBS, treated with the indicated agents for 24 h and incubated in medium containing 3% FBS for another 24 h. The cells were collected and resuspended in 100 μL of PBS, sonicated on ice and collected the supernatants after centrifugation at 1500×*g* for 10 min at 4 °C. Protein concentrations were determined by Coomassie Plus Protein Assay Reagent (Thermo Fisher Scientific). The samples were mixed with the enzyme working solution and chromogenic agent and incubated at 37 °C for 10 min. The stop solution was added, and the absorbance was measured at 530 nm by a microplate reader (TECAN). LA levels were calculated by the following formula: LA (mmol/gm protein) = (ΔOD1 sample/ΔOD2 standard) × 3 (the concentration of standard, mmol/l) × dilution factor of sample/concentration of protein in sample (gm of protein/l).

### 4T1 TNBC progression, metastasis and recurrence

Six-week-old female BALB/c mice were obtained from the National Laboratory Animal Center (Taipei, Taiwan) and approved by the Animal Use Protocol (NHRI-IACUC-108026-A). 4T1 cancer cells (1 × 10^4^) were implanted in the 4th mammary fat pad. Tumor volume was measured weekly as previously described [34].

To evaluate postsurgical therapeutic outcomes, primary tumors were surgically removed when the tumor volumes reached approximately 150 mm^3^ (week 2). Xenograft mice were intraperitoneally injected with olaparib (50 mg/kg) twice per week for 2 weeks or were administered Oligo-Fucoidan (150 mg/kg) by oral feeding twice per week for 5 weeks. PBS was used as a control treatment. At week 7, tumor relapse, metastasis and mouse mortality rates were evaluated. Recurrent and metastatic tumors were processed for further analysis as previously described [34].

### Immunohistochemical (IHC) analysis

IHC analysis of tumors was performed using a DAKO EnVision detection kit (Agilent Technologies, Santa Clara, CA, USA). Anti-CD163 Ab (E-AB-70306) (Elabscience Biotechnology Inc, Houston, TX, USA) (1:600) was added and incubated with the specimens for 30 min, after which the samples were washed with PBS for 15 min and incubated with a labeled polymer-HRP-conjugated secondary Ab (DAKO EnVision System) for 10 min. The Ab-reactive samples were visualized using 3,3′-diaminobenzidine substrate (DAKO EnVision System), counterstained with Mayer’s hematoxylin (Sigma–Aldrich), dehydrated with ethanol (70%, 3 min; 80%, 3 min; 95%, 3 min; and 100%, 5 min), cleared with xylene (5 min), and cover-slipped (Automat-star 24 × 50 mm) with Histokitt solution mounting medium (Glaswarenfabrik Karl Hecht GmbH & CO KG., Germany). IHC images were observed under a Nikon Optiphot-2 upright microscope with a 40 × objective lens and analyzed by the automatic digital slide scanner system Pannoramic MIDI II (3DHISTECH, Ltd., Budapest, Hungary). ImageJ Fiji software (version 1.2) (NIH, Bethesda, MD, USA) was used to conduct color deconvolution to adjust the image threshold and analyze the signal intensity as described previously [35]. The results were evaluated in at least 6 randomly chosen fields.

### Flow cytometric analysis of splenic lymphocytes

The olaparib and/or Oligo-Fucoidan-treated 4T1 cell xenograft mice were sacrificed at week 7. The spleen tissue was harvested and rinsed by PBS, gently minced in ice-cold RPMI-1640 (Life Technologies) containing 2% FBS (Life Technologies), and then mashed using the plunger of a 1 ml syringe. The cell suspension was passed through a 70-μm-nylon cell strainer to remove clumps, lysed with red blood cell lysis buffer (155 mM NH_4_Cl, 12 mM NaHCO_3_ and 0.1 mM EDTA), and fixed with 4% (v/v) paraformaldehyde (Alfa Aesar, Ward Hill, MA, USA). The cell suspension (1 × 10^6^) was then resuspended with anti-mouse CD16/CD32 Ab (1:2000) in 0.1% bovine serum albumin (Sigma–Aldrich) in ice-cold PBS to block nonspecific binding and labeled with cocktails of biotin-conjugated mouse anti-mouse NK-1.1, rat anti-mouse CD19 Ab, rat anti-mouse CD4 Ab, and rat anti-mouse CD8a Ab at a 1:100 dilution. The labeled cells were purified by positive selection using Streptavidin Particles Plus. Purified lymphocytes were stained with PE mouse anti-mouse NK-1.1 Ab (1:25), APC rat anti-mouse CD19 Ab (1:50), PerCP-Cy5.5 rat anti-mouse CD4 Ab (1:50) and BB515 rat anti-mouse CD8a Ab (1:100). The isotype control Abs of PE hamster IgG2 κ, BB515 rat IgG2b κ, APC rat IgG2a κ and PerCP-Cy5.5 rat IgG2a κ were used as negative controls. All the biotinylated and conjugated Abs used for flow cytometry analysis of the lymphocytes were purchased from BD Biosciences. Analysis was performed using the BD FACSCalibur flow cytometer and FlowJo v10. BB515 rat anti-mouse CD8a Ab, PE mouse anti-mouse NK-1.1 Ab and PerCP-Cy5.5 rat anti-mouse CD4 Ab were excited by a 488-nm laser line, and the emissions were measured in the FL1 channel (515–545 nm) with a 530/30 BP filter, FL2 channel (564–606 nm) with a 585/42 bandpass (BP) filter and FL3 channel with a 670 nm long-pass (LP) filter, respectively. APC rat anti-mouse CD19 Ab was excited by a 635-nm laser line, and the emission was measured in the FL4 channel (653–669 nm) with a 661/16 BP filter.

### Flow cytometric analysis of tumor-infiltrating Tregs

Recurrent breast tumor tissues were minced and enzymatically digested with a cocktail containing 1 mg/ml collagenase D (Sigma–Aldrich), 0.25 mg/ml DNase I (Sigma–Aldrich), and 0.25% (v/v) trypsin–EDTA solution (HyClone, Logan, UT, USA) in serum-free RPMI-1640 medium (Life Technologies). Similar with lymphocyte preparation, a single-cell suspension (1 × 10^6^) was blocked with anti-mouse CD16/CD32 (1: 2000) (Sigma–Aldrich) and then positively selected by anti-mouse CD4 magnetic particles and then stained with PerCP-Cy5.5 rat anti-mouse CD4 Ab (1:50), PE rat anti-mouse CD25 Ab (1:25) and APC rat anti-mouse CD127 Ab (1:50). The isotype controls Abs (PE hamster IgG2 κ, APC rat IgG2a κ and PerCP-Cy5.5 rat IgG2a κ) were used as negative controls. Analysis was performed using the BD FACSCalibur flow cytometer and FlowJo v10. Tregs were defined as CD25(+)/CD127(−) subsets gated from CD4(+) populations. PE rat anti-mouse CD25 Ab and PerCP-Cy5.5 rat anti-mouse CD4 Ab were excited by a 488-nm laser line, and the emissions were measured in the FL2 channel (564–606 nm) with a 585/42 BP filter and the FL3 channel with a 670 nm LP filter, respectively. APC rat anti-mouse CD127 Ab was excited by a 635-nm laser line, and the emission was measured in the FL4 channel (653–669 nm) with a 661/16 BP filter. All the conjugated Abs were purchased from BD Biosciences.

### Statistics

The data are presented as the means ± S.E.M. One-way ANOVA with Newman–Keuls test or Duncan’s test was used to analyze the data. ^*^p < 0.05, ^**^p < 0.01, ^***^p < 0.001.

## Results

### Olaparib and Oligo-Fucoidan cooperatively induce TNBC cell death

**Fig. 1:**
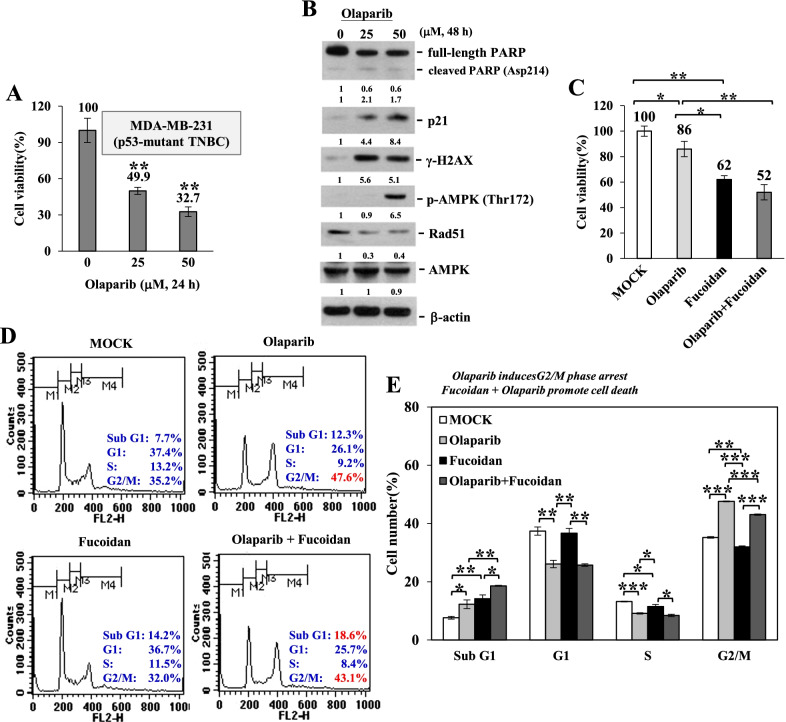
Olaparib and Oligo-Fucoidan cooperatively promote TNBC cell death. **A** Cell viability was examined following olaparib (0–50 μM) treatment for 24 h and was examined by WST-1 assays. **B** The levels of PARP, cleaved PARP (Asp214), p21, γ-H2AX, p-AMPK (Thr172), AMPK and Rad51 were analyzed after olaparib (0–50 μM) treatment for 48 h. **C** Cell viability was analyzed by WST-1 assays after olaparib (50 μM) and/or Oligo-Fucoidan (400 μg/ml) treatment for 24 h. **D** Cell cycle profiling was performed after olaparib (50 μM) and/or Oligo-Fucoidan (400 μg/ml) administration for 48 h. **E** Histograms reveal the cell cycle patterns and comparisons between experimental settings. One-way ANOVA with Duncan’s test was used to calculate statistical significance (**A, C, E**)

Olaparib is an excellent PARP inhibitor that blocks DNA damage repair and induces cell death by synthetic lethality in BRCA1/2-mutated carriers [36]. We found that olaparib treatment decreased the viability of p53-mutant and BRCA1-deficient TNBC MDA-MB-231 cells in a dose-dependent manner (Fig. 1A). Consistently, the cleaved PARP (Asp214), p21 (cyclin-dependent kinase inhibitor), γ-H2AX (DNA double-strand break biomarker) and p-AMPK (Thr172) (energy depletion sensor) were induced but the full-length PARP and Rad51 (DNA repair protein) were reduced by olaparib treatment in a dose-dependent manner (Fig. 1B).

Cell viability was analyzed after olaparib and/or Oligo-Fucoidan treatment, and the results show that Oligo-Fucoidan combined with olaparib more suppressed the survival of MDA-MB-231 cells than individual effects (Fig. 1C). Further analysis of the cell cycle profile showed that G2/M arrest was induced by olaparib (47.6%) but not Oligo-Fucoidan (32%) compared with MOCK treatment (35.2%) (Fig. 1D, E). Sub-G1 apoptotic populations were moderately increased by olaparib (12.3%) and Oligo-Fucoidan (14.2%) relative to MOCK treatment (7.7%), and combination treatment further enriched the sub-G1 populations (18.6%) than individual treatments. Consequently, olaparib and Oligo-Fucoidan combined significantly diminish TNBC cell viability.

### Combined olaparib and Oligo-Fucoidan treatment inhibits TNBC cell growth and stemness

**Fig. 2:**
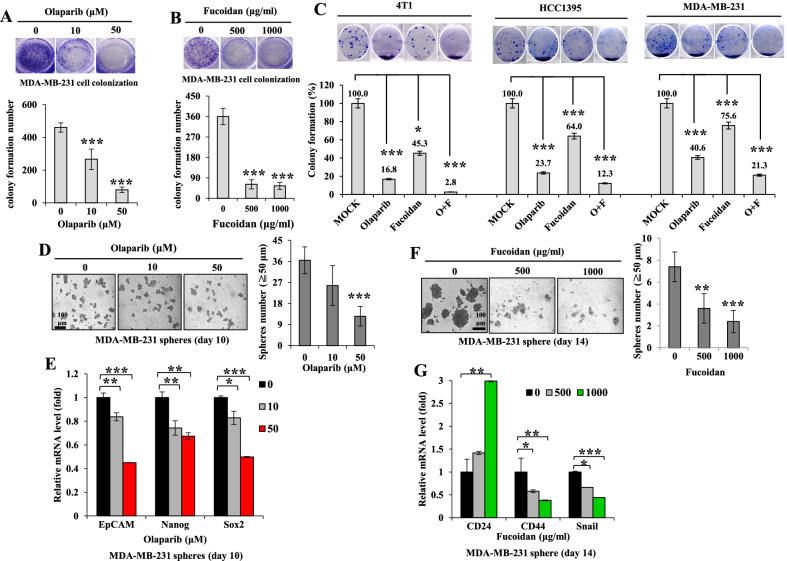
Olaparib combined with Oligo-Fucoidan further inhibits TNBC cell growth and stemness properties. **A, B** Clonogenic formation of MDA-MB-231 cells was examined after treatment with different doses of olaparib (**A**) or Oligo-Fucoidan (**B**) for 2 weeks (n = 6). **C** The colony formation capacities of 4T1, HCC1395 and MDA-MB-231 cells were examined after olaparib (50 μM) and/or Oligo-Fucoidan (400 μg/ml) treatment for 2 weeks (n = 6). **D–E** MDA-MB-231 mammosphere formation (≥ 50 µm in diameter) and the expression levels of cancer stemness markers (EpCAM, Nanog and Sox2) were measured by qRT–PCR on Day 10 in response to olaparib (0–50 μM) treatment (n = 5). **F–G** MDA-MB-231 mammosphere formation (≥ 50 µm in diameter) and the expression levels of cancer stemness markers (CD24, CD44 and Snail) were measured by qRT–PCR on Day 10 in response to Oligo-Fucoidan (0–1000 μg/ml) treatment for 2 weeks (n = 5). The data represent the mean ± SD. One-way ANOVA with Duncan’s test was used to define statistical significance

We next performed clonogenic formation assays and found that MDA-MB-231 cell growth was significantly prevented by olaparib (Fig. 2A) and by Oligo-Fucoidan (Fig. 2B) administration in a dose-dependent manner. The combination of olaparib and Oligo-Fucoidan resulted in higher inhibition of cell colony formation than monotherapy in different TNBC cell lines (4T1, HCC1395 and MDA-MB-231) (Fig. 2C). To assess breast cancer stem cell (BCSC) properties, MDA-MB-231 cells form mammospheres in stem cell culture condition on a low-attachment plate. Olaparib treatment not only inhibited the development of mammospheroids (Fig. 2D) but also downregulated the expression levels of cancer stemness markers (Fig. 2E), such as EpCAM, Nanog and Sox2. Similarly, Oligo-Fucoidan treatment prevented mammosphere formation in a dose-dependent manner (Fig. 2F) and reduced BCSC features that upregulated CD24 and downregulated CD44 and Snail expression (Fig. 2G).

**Fig. 3:**
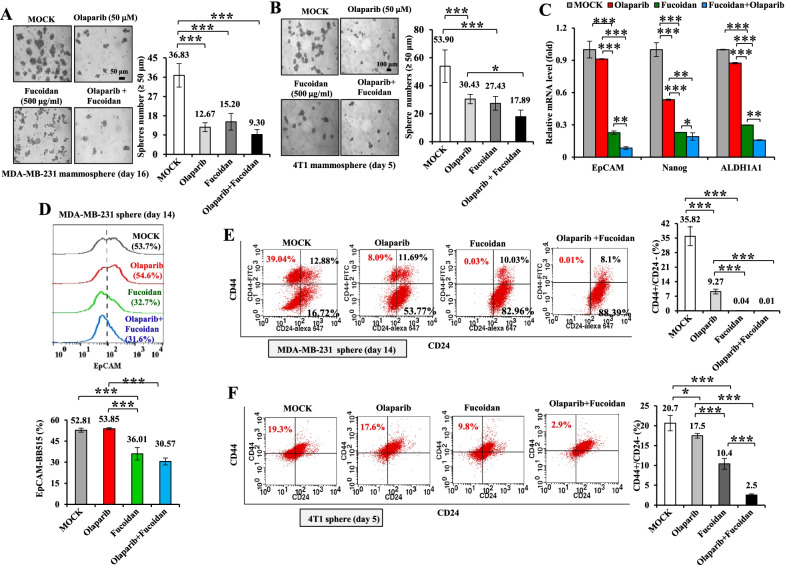
Olaparib and Oligo-Fucoidan synergistically inhibit TNBC cell stemness. **A**, **B** Mammosphere formation abilities of MDA-MB-231 cells (**A**) (n = 6–10) and 4T1 cells (**B**) (n = 7–10) were analyzed in response to olaparib (50 μM) and/or Oligo-Fucoidan (500 μg/ml) treatment for 16 and 5 days, respectively. **C** The mRNA expression levels of EpCAM, Nanog and ALDH1A1 in MDA-MB-231 mammospheres (Day 16) were quantified by qRT–PCR (n = 5). **D** EpCAM(+) subpopulations in MDA-MB-231 mammospheres (Day 14) were examined by flow cytometry after the indicated treatments (n = 4–5). **E**, **F** CD44(+)/CD24(−) subpopulations were analyzed in MDA-MB-231 mammospheres (Day 14) (**E**) and 4T1 mammospheres (Day 5) (**F**) after the indicated treatments. The data are expressed as the mean ± SD. One-way ANOVA with Duncan’s test was used to determine statistical significance

**Fig. 4:**
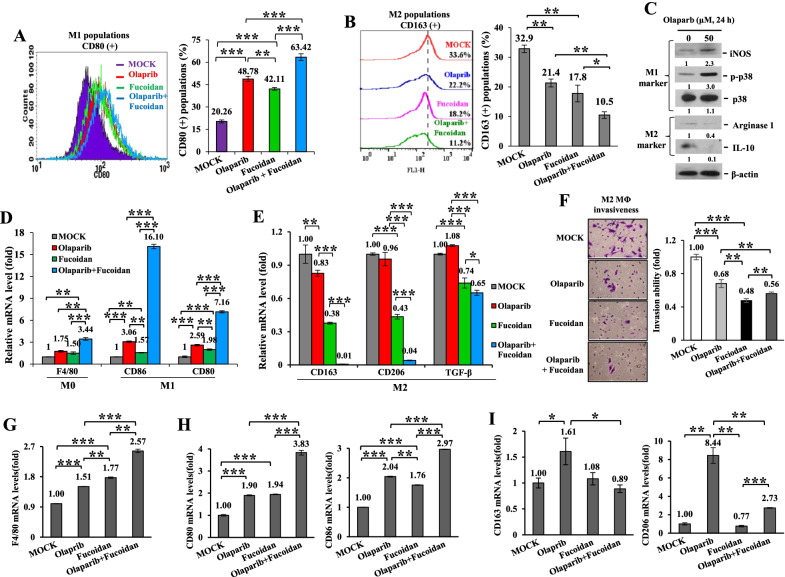
Olaparib plus Oligo-Fucoidan is superior to monotherapy in inhibiting M2 macrophage plasticity and invasiveness. **A**, **B** THP-1 monocytes were treated with olaparib (50 μM) and/or Oligo-Fucoidan (400 μg/ml) for 48 h. The populations of CD80(+) M1 (**A**) and CD163(+) M2 (**B**) macrophages were evaluated by flow cytometry (n = 5). **C** Intracellular markers of M1 (iNOS and p-p38) and M2 (Arginase 1 and IL-10) macrophages were compared before and after olaparib (50 μM) treatment of THP-1 monocytes for 24 h. **D**, **E** The expression levels of M0 (F4/80), M1 (CD80, CD86) (n = 5) (**D**) and M2 (CD163, CD206 and TGF-β) (n = 5) (**E**) markers in response to the indicated treatments were evaluated by qRT–PCR. **F–I** THP-1 monocytes were pretreated with PMA (100 ng/ml) for 24 h and IL-4 (50 ng/ml) for another 24 h to activate differentiation of M2 macrophages. M2 invasion abilities (n = 3) (**F**) and the expression levels of M0 (F4/80) (n = 5) (**G**), M1 (CD80 and CD86) (n = 5) (**H**) and M2 (CD163 and CD206) (n = 5) (**I**) markers were analyzed after the indicated treatments for 48 h. The data are expressed as the mean ± SD. One-way ANOVA with Duncan’s test was used to estimate statistical significance

Further analysis of the combined effects of olaparib and Oligo-Fucoidan on BCSC properties, the results showed that both agents effectively suppressed mammosphere formation by MDA-MB-231 cells (Day 16) (Fig. 3A) and 4T1 cells (Day 5) (Fig. 3B), and combined treatment did not result in further suppression. However, the expression levels of cancer stemness markers (EpCAM, Nanog and ALDH1A1) were much suppressed in MDA-MB-231 mammospheres (Day 14) in response to Oligo-Fucoidan or the combined treatment than olaparib alone (Fig. 3C). Similarly, the transmembrane glycoprotein EpCAM on cancer stem cells was analyzed by flow cytometry and indicated that the EpCAM(+) subpopulations in MDA-MB-231 mammospheres (Day 14) were not inhibited by olaparib alone but significantly suppressed by Oligo-Fucoidan and the combined treatment (Fig. 3D). When CD24(−)/CD44(+) subpopulations in MDA-MB-231 mammospheres (Day 14) (Fig. 3E) and 4T1 mammospheres (Day 5) (Fig. 3F) were examined, Oligo-Fucoidan alone and in combination with olaparib also decreased CD24(−)/CD44(+) cells more than olaparib alone. Therefore, combined Oligo-Fucoidan and olaparib effects additively reduce TNBC stem cell properties.

### Olaparib and Oligo-Fucoidan cooperatively prevent M2 macrophage differentiation and invasion

To analyze macrophage polarity in vitro, human THP-1 monocytes were directly treated with olaparib and/or Oligo-Fucoidan. Surface markers of polarized macrophages were examined by flow cytometry and revealed that CD80(+) M1 populations were induced by olaparib (48.78%) and Oligo-Fucoidan (42.11%), which were further enhanced by combination treatment (63.42%) compared with MOCK treatment (20.26%) (Fig. 4A). In contrast, CD163(+) M2 macrophage populations were repressed, particularly by the combined treatment (10.5%) compared with MOCK treatment (32.9%) and olaparib (21.4%) or Oligo-Fucoidan (17.8%) alone (Fig. 4B). Intracellular M1 macrophage markers (iNOS and p-p38 MAPK (Thr180/Tyr182)) were increased, but M2 markers (Arginase 1 and IL-10) were decreased in olaparib-treated THP-1 cells (Fig. 4C). Also, the expression levels of M0 (F4/80) and M1 (CD86 and CD80) macrophage markers were induced by olaparib or Oligo-Fucoidan alone, and they were further advanced by the combined therapy (Fig. 4D). Although olaparib alone incapably reduced the expression of M2 markers (CD206 and TGF-β), Oligo-Fucoidan combined with olaparib significantly inhibited these markers and also CD163 expression (Fig. 4E).

To analyze the M2 macrophage response, THP-1 monocytes were stimulated with PMA and then IL-4. The M2 macrophages were then treated with olaparib and/or Oligo-Fucoidan, and their invasion abilities were repressed (Fig. 4F), while M0 (F4/80) (Fig. 4G) and M1 (CD80 and CD86) (Fig. 4H) markers were upregulated. Unexpectedly, the olaparib-treated M2 macrophages further induced the expression of M2 markers (CD163 and CD206), but these unfavorable effects were significantly repressed by Oligo-Fucoidan supplementation (Fig.4I).

### Combining Olaparib and Oligo-Fucoidan treatment of TNBC cells further inhibits the oncogenic pathway and glucose metabolism and results in M1 macrophage polarization

**Fig. 5:**
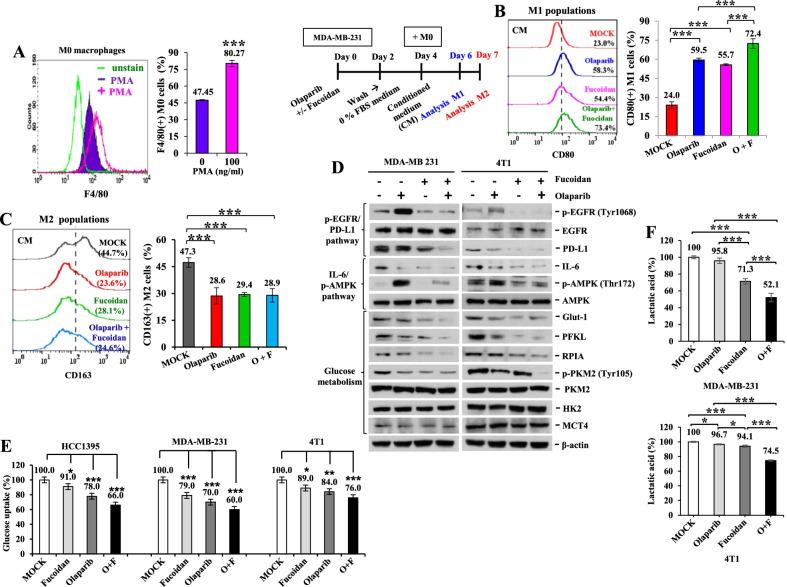
Olaparib- and Oligo-Fucoidan-treated TNBC cells promote M1 macrophage plasticity and inhibit the oncogenic and glycolytic pathways. **A** The F4/80(+) M0 macrophage population was examined by flow cytometry (n = 6) after THP-1 monocytes were treated with PMA (100 ng/ml) for 72 h. **B**, **C** MDA-MB-231 cells were pretreated with olaparib (50 μM) and/or Oligo-Fucoidan (400 μg/ml) for 48 h and cultured in serum-free medium for 48 h, after which the conditioned medium (CM) was collected and incubated with M0 macrophages for 48 h and 72 h to examine the polarity of CD80(+) M1 (**B**) and CD163(+) M2 macrophages (**C**) by flow cytometry (n = 6). **D** IL-6, EGFR/p-EGFR (Tyr1068), AMPK/p-AMPK (Thr172), PD-L1 and glucose metabolism factors (Glut1, PFKL, RPIA, PKM2/p-PKM2 (Tyr105), HK2, MCT4) were examined in MAD-MB-231 and 4T1 cells after olaparib (50 μM) and/or Oligo-Fucoidan (400 μg/ml) treatment for 24 h. **E**, **F** Glycolytic pathways, such as glucose uptake (**E**) and lactate production (**F**), were analyzed in TNBC cell lines after the indicated treatments for 24 h (n = 4). The data represent the mean ± SD. Student’s t test (**A**) and one-way ANOVA with Duncan’s test (**B**, **C**, **E**, **F**) were used to calculate statistical significance

To assess the impact of the treated TNBC cells on macrophage plasticity, THP-1 monocytes were administered PMA to induce polarization of F4/80(+) M0 macrophages (Fig. 5A). After MDA-MB-231 cells were pretreated with olaparib and/or Oligo-Fucoidan, M0 macrophages were cultured with the resulting conditioned media (CM). The CM of TNBC cells that were pretreated with olaparib (O) (59.45%), Oligo-Fucoidan (F) (55.72%) and the combination (O + F) (72.42%) induced CD80(+) M1 macrophage polarity compared with MOCK treatment (23.0%) (Fig. 5B). In contrast, all CM from olaparib- and/or Oligo-Fucoidan-pretreated TNBC cells successfully blocked the polarization of M0 macrophages to CD163(+) M2 macrophages (Fig. 5C). These could be due to the factors released from the TNBC cells have been significantly suppressed that prevent M2 macrophage function.

We next analyzed whether olaparib and/or Oligo-Fucoidan could inhibit the oncogenic pathway in different TNBC cell lines. Although the phosphorylation of the survival kinase EGFR (p-EGFR Tyr1068) in MDA-MB-231 or 4T1 cells were induced by olaparib, this hostile effect was significantly suppressed by Oligo-Fucoidan alone and combination treatment (Fig. 5D). Corresponding to EGFR deactivation, the inflammatory cytokine IL-6 and the immune checkpoint protein PD-L1 were also suppressed by olaparib plus Oligo-Fucoidan. PD-L1 mediates immune evasion of aggressive cancers [37]. AMPK activation induces the phosphorylation of PD-L1 that causes PD-L1 degradation [38]. However, independent of AMPK activity, olaparib plus Oligo-Fucoidan reduced PD-L1 expression and AMPK activation, suggesting that the immune surveillance can be improved by combination treatment via blocking the IL-6/EGFR/PD-L1 pathway. Similar to the effect of the EGFR inhibitor (DBPR112) (Institute of Biotechnology and Pharmaceutical Research, National Health Research Institutes (NHRI), Taiwan), treatment with Oligo-Fucoidan and olaparib reduced PD-L1 levels but not the effect of anti-PD-L1 immunotherapy (Atezolizumab) (Selleckchem, Houston, TX, USA) in HCC1395 cells (Additional file 1: Fig. S1).

AMPK, a sensor of glucose limitations and cellular energy status [39, 40], is activated to resolve nutrient resource–need imbalances and is critical for diabetes and cancer development. IL-6 acts on glucose metabolism [41] and AMPK activation to promote GLUT4 translocation to the neuronal plasma membrane [42], resulting in enhanced glucose uptake in neuronal cells. Relevant to the inhibition of the IL-6/p-AMPK axis by combined olaparib and Oligo-Fucoidan treatment, we found that not only glucose metabolism factors, such as glucose transporter-1 (Glut1), phosphofructokinase (PFKL), ribose 5-phosphate isomerase A (RPIA) and phosphor-pyruvate kinase 2 (p-PKM2) (Tyr105) (Fig. 5D) but also glucose uptake (Fig. 5E) were all substantially decreased in different TNBC cell lines (MDA-MB-231 and 4T1).

The end metabolite of anaerobic or aerobic glycolysis in cancer cells is lactate, which induces the expression of vascular endothelial growth factor and the polarization of M2-like TAMs [43]. Large amounts of lactate cause an acidic TME that supports tumor metastasis, angiogenesis and immunosuppression [44], which result in a worse clinical prognosis. We found that lactic acid levels in MDA-MB-231 and 4T1 cells were also suppressed in response to combined olaparib and Oligo-Fucoidan treatment compared to monotherapy (Fig. 5F), which may stop the feeding of cancer cells and stromal cells by circulating lactate to avoid acidosis TME, immune evasion and cancer stemness.

### Oligo-Fucoidan plus olaparib efficiently inhibits postsurgical TNBC metastasis and relapse

**Fig. 6:**
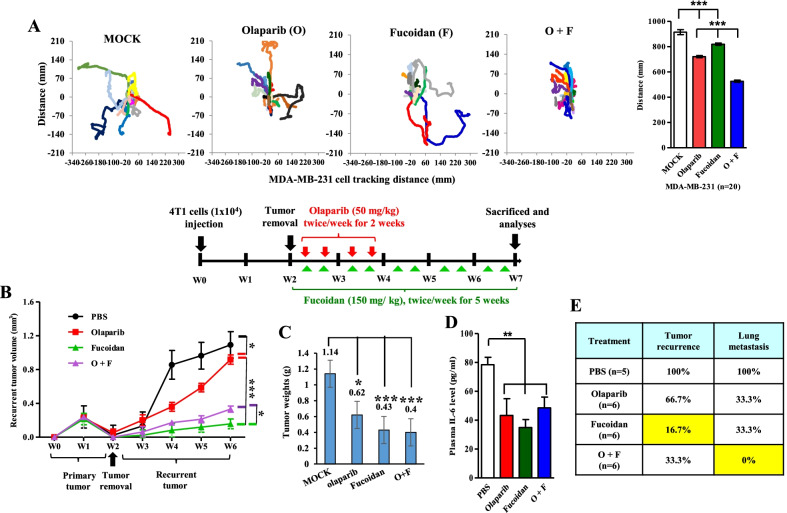
Oligo-Fucoidan combined with olaparib therapy better prevent TNBC aggressiveness than olaparib alone. **A** The trajectories of 4T1 cell migration were analyzed and compared after olaparib (50 μM) and/or Oligo-Fucoidan (400 μg/ml) treatment for 48 h (n = 20). **B–E** 4T1 cells (1 × 10^4^) were orthotopically inoculated into BALB/c mice, and the primary breast tumors were surgically removed at week 2 followed by olaparib (50 mg/kg) treatment via i.p. injection twice per week for 2 weeks or combined with Fucoidan (150 mg/kg) by oral feeding twice per week for 5 weeks (n = 6). PBS was used as the control treatment (n = 5). The volumes of primary mammary tumors and the recurred breast tumors in response to the indicated treatments were examined (**B**). At the endpoints of the indicated treatments (week 7), the recurrent tumor loads (**C**) and plasma IL-6 levels (**D**) were measured. The incidences of postsurgical mammary tumor relapse and distant metastasis indicated the therapeutic efficacies (**E**). The data are presented as the means ± S.E.M. One-way ANOVA and Newman–Keuls test were used to analyze the data

Murine 4T1 tumor progression resembles features of advanced human TNBC, including a high degree of distant metastasis and local recurrence [45, 46]. We compared the effects of combined and individual therapies on the mobility of 4T1 cells, and the results showed that olaparib (O) and/or Oligo-Fucoidan (F) treatment effectively restricted the trajectories of cell migration (Fig. 6A), suggesting that TNBC aggressiveness will be inhibited.

To further analyze the anti-TNBC therapeutic effects, the mammary fat pads of BALB/c mice were injected with 4T1 cells (1 × 10^4^) (W0). The resulting mammary tumors that developed in xenograft mice were surgically removed at week 2 (W2), and the primary tumor sizes reached approximately 100–150 mm^3^. Starting on postsurgery Day 3, olaparib was administered intraperitoneally (i.p.) twice weekly for 2 weeks, and/or Oligo-Fucoidan was orally administered to the mice twice weekly for 5 weeks. The results showed that Oligo-Fucoidan alone and in combination of olaparib had a greater suppressive effect on 4T1 TNBC progression than olaparib alone (Fig. 6B). The average tumor burdens also indicated that postsurgical relapse at week 7 was significantly reduced by olaparib (0.62 g), Oligo-Fucoidan (0.43 g) and the combination treatment (0.4 g) compared with the PBS control (1.14 g) (Fig. 6C). Serum levels of IL-6, which are associated with tumor progression, were also reduced by olaparib and/or Fucoidan treatment at week 7 (Fig. 6D). Postoperative treatment with olaparib alone decreased the tumor relapse (66.7%) and substantially inhibited lung metastasis (33.3%) compared with the PBS control (100%). Surprisingly, Oligo-Fucoidan alone also had a robust impact on preventing local recurrence (16.7%) and lung metastasis (33.3%) of TNBC (Fig. 6E), and it reduced the postsurgical recurrence (33.3%) and stopped the predominant lung metastasis (0%) when combined with olaparib. Therefore, after surgical removal of the primary TNBC, incidences of local recurrence and lung metastasis were more effectively repressed by Oligo-Fucoidan and plus olaparib therapy than olaparib alone.

### Antitumor immunity is improved by Oligo-Fucoidan and olaparib combined therapy

**Fig. 7:**
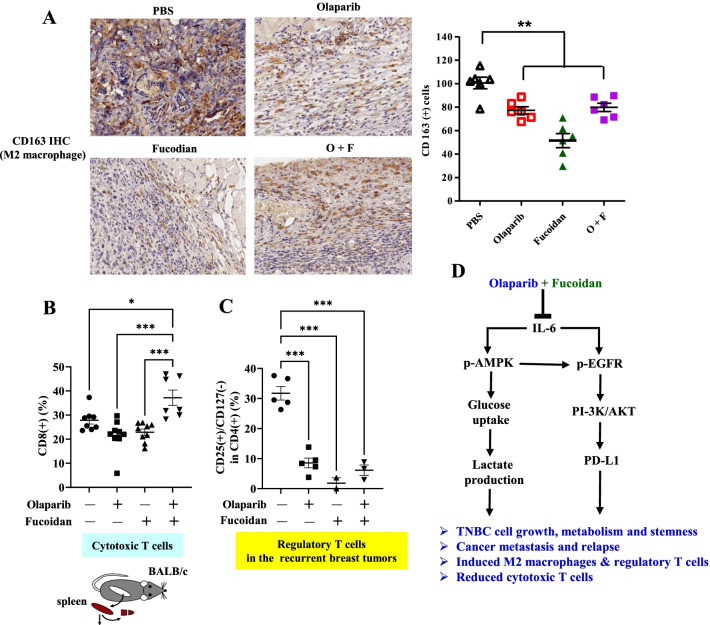
Olaparib plus Oligo-Fucoidan improves anti-TNBC immunity. Splenic immune cells, tumor-associated macrophages and T cells were analyzed in postsurgical mice bearing TNBC and treated with olaparib (50 mg/kg) and/or Oligo-Fucoidan (150 mg/kg). **A** Immunohistochemical analysis of CD163(+) M2 macrophages in recurrent mammary tumors. **B**, **C** Flow cytometry analysis of CD8( +) cytotoxic T cells in spleen (**B**) and CD4(+)/CD25(+)/CD127(−) Tregs in recurrent breast tumor after the indicated treatments (**C**). One-way ANOVA with Duncan’s test was used to calculate statistical significance. **D** Summary diagram of the effects of olaparib plus Oligo-Fucoidan treatment on suppressing the oncogenic IL-6/p-EGFR/PD-L1 pathway and AMPK activation, which impact TNBC cell growth, stemness and metabolism. The combined treatment prevents TNBC relapse and metastasis, as well as immune evasion, by decreasing immunosuppressive M2 macrophages and Tregs and increasing cytotoxic T cells

We measured M2 macrophages in recurrent 4T1 mammary tumors by immunohistochemistry. The quantities of intratumoral CD163(+) M2 macrophages were reduced by Oligo-Fucoidan compared with olaparib and the combined treatment (Fig. 7A). Flow cytometry analysis of splenic immune cells after the indicated treatments further revealed that CD8(+) cytotoxic T cells were significantly increased only when the postoperative mice were administered with olaparib plus Oligo-Fucoidan (Fig. 7B), but CD4(+) helper T cells, natural killer (NK)1.1(+) cells and CD19(+) B cells were not affected by the indicated treatments (Additional file 1: Fig. S2B) compared with isotype control Abs for flow cytometric evaluation (Additional file 1: Fig. S2A). Importantly, in recurrent mammary tumors, the quantities of CD4(+)/CD25(+)/CD127(−) regulatory T cells (Tregs) present were significantly diminished following postsurgical treatment with olaparib and/or Oligo-Fucoidan (Fig. 7C). Accordingly, immune surveillances in lymphatic system and in TME of mice bearing TNBC responds differently to olaparib and/or Oligo-Fucoidan therapy.

## Discussion

The DNA repair enzyme PARP has been recognized as a promising therapeutic target for the treatment of tumor malignancies [47], particularly those associated with dysfunctional DNA repair pathways such as TNBCs. To our knowledge, this is the first preclinical study to examine in detail the therapeutic effectiveness of natural compound Oligo-Fucoidan plus olaparib in treating invasive TNBC. We demonstrated for the first time that Oligo-Fucoidan potently enhanced immune surveillance in metastatic TNBC in mice and inhibited the EGFR/PD-1 signaling in TNBC cells. Supplementing olaparib therapy with Oligo-Fucoidan reprogrammed the plasticity of innate and adaptive immune cells, which could reprogram the microenvironment against TNBC stemness, metabolism, recurrence and metastasis (Fig. 7D). The immune cells in the lymphatic system and TME were further analyzed to predict anti-TNBC immunity in mice, and the results showed that Oligo-Fucoidan more effectively reduced infiltration of immunosuppressive M2 macrophages and Tregs in recurrent TNBC than olaparib alone (Fig. 7A, C). Oligo-Fucoidan plus olaparib also efficiently increased cytotoxic T cells in the lymphoid system (Fig. 7B), suggesting that the combination therapy will impede postsurgical TNBC regression and enhance overall survival outcomes. Therefore, the right timing for tumor resection, drug delivery and appropriate supplementation will determine therapeutic perspectives and can offer a safe and more effective approach to overcome tumor heterogeneity and aggressiveness with fewer adverse effects of chemotherapy.

Recruitment of M2 macrophages impacts tumor relapse and lymph node metastasis after chemotherapy [48], determining the poor prognosis of patients. Oligo-Fucoidan and olaparib was found to cooperatively affect THP-1 monocytes better than monotherapy, improving M1 polarization and inhibiting M2 invasion and polarity (Fig. 4). Furthermore, these therapies decreased inflammatory IL-6 levels of postsurgical TNBC-bearing mice (Fig. 6D); thus, the treated TNBC cells had diminished secretion of the factor(s) that favor activated M2 macrophage functions (Fig. 5B, C). Oligo-Fucoidan and olaparib modulate macrophage plasticity and infiltration via paracrine and/or autocrine mechanisms could boost antitumor immunity.

PD-L1 is induced in TNBCs [49]; thus, anti-PD1/PD-L1 immunotherapies are recommended for TNBC treatment [50], either by delivery as a single or combination therapy. AMPK activation directly phosphorylates PD-L1 (S195) and triggers abnormal PD-L1 glycosylation and endoplasmic-reticulum-associated degradation of PD-L1 [38], which promotes the immune surveillance of CD8(+) cytotoxic T cells and suppresses breast tumor progression. Here, we showed that the IL-6/p-EGFR/p-AMPK/PD-L1 axis was associated with the promotion of TNBC aggressiveness and stemness and the immunosuppressive TME. However, immune escape via the p-AMPK/PD-L1 network in TNBC cells could be reversed by systemic treatment with Oligo-Fucoidan and olaparib (Fig. 5D). In addition, Oligo-Fucoidan and olaparib together further reduced oncogenic IL-6/p-EGFR signaling, which may downregulate PD-L1 and impair the PD-L1/PD-1 interaction between TNBC cells and T effector cells, resulting in the proliferation of cytotoxic T cells to enhance therapeutic efficacy. Consistently, TNBC immune evasion could be inhibited by olaparib and Oligo-Fucoidan combination (Fig. 7B, C); thus, the exhaustion of CD4^+^ helper T cells and CD8^+^ cytotoxic T cells may decrease.

Glycolytic enzymes, such as hexokinase 2 (HK2) and PKM2 isoenzyme, are highly expressed in TNBC cells and mediate glycolysis and tumor development [51, 52], which activate NF-kB and its downstream target genes [53]. Phosphorylated PKM2 (Tyr105) is oncogenic and promotes cancer stemness properties [54]. Lactate dehydrogenase A (LDHA) catalyzes the conversion of pyruvate to lactate in the cytoplasm. TNBC cells exhibit higher LDHA and AMPK levels and lower oxygen consumption rates than luminal breast cancer cells [55]. Monocarboxylate transporters (MCTs) control lactate efflux and TNBC cell proliferation, migration and tumor growth [56]. The accumulation of glycolytic intermediates such as lactate provide proliferative advantages for TNBC expansion and evade destruction by cytotoxic T cells [57]. Therefore, suppressing glycolysis-related molecules by Oligo-Fucoidan supplement could help TNBC treatment.

Glucose uptake induces PD-L1 expression and PD-L1-mediated immune escape in the malignant kidney tumor microenvironment via the EGFR/ERK/c-Jun pathway [37]. The combination of an EGFR inhibitor, gefitinib and the glycolysis inhibitor 2-deoxy-D-glucose has been shown to effectively prevent TNBC progression [57]. The combined effects of Oligo-Fucoidan and olaparib suppressed glucose uptake and lactate production (Fig. 5E, F) and decreased Glut-1, PFKL, RPIA and p-PKM2 and the IL-6/p-EGFR/p-AMPK/PD-L1 network (Fig. 5D), which can reprogram cancer cell energy expenditure and the PD-L1/PD-1 interaction between TNBC cells and T effector cells, enhancing immune checkpoint control.

## Conclusion

Our findings open a new therapeutic direction that could have an important impact on the management of metastatic TNBCs. Anti-PD-L1/PD-1 or anti-IL-6/IL-6 receptor (IL-6R) immunotherapies cooperative with Oligo-Fucoidan will be further examined to determine whether they effectively enhance current therapeutics and immunotherapies.

## Supplementary Information

**Additional file 1: Fig. S1.** PD-L1 is suppressed by DBPR112, Oligo-Fucoidan and olaparib. PD-L1 levels in HCC1395 cells were examined after treatment with DBPR112 (5 ng/ml), Oligo-Fucoidan (400 μg/ml), olaparib (50 μM) or anti-PD-L1 (50 μM) for 48 h. **Fig. S2. **Olaparib and Oligo-Fucoidan treatment do not affect CD4(+) T cells, B cells or NK cells in lymphoid system. **(A) **Representative FACS plots showing the gating strategy for the immunophenotyping of splenic T-, B- and NK-cell subpopulations. Cytotoxic T cells, T helper cells, B cells and NK cells were separated from the lymphocyte gate via CD8(+) vs. side scatter (SSC), CD4(+) vs. SSC, CD19(+) vs. SSC and NK1.1(+) vs. SSC, respectively. Analysis of CD4(+) **(B)**, NK1.1(+) **(C) **and CD19(+) **(D) **subpopulations showed that neither monotherapy nor dual treatment with olaparib and Oligo-Fucoidan affected the abundance of CD4(+) T helper cells, B cells, or NK cells. Vehicle (PBS), n=8; olaparib, n=9; Fucoidan, n=9; olaparib and Fucoidan combination, n=7. **(E)** Representative FACS plots delineating recurrent tumor-infiltrating Tregs. Tregs were identified by CD25(+) and CD127(-) staining of CD4(+) cells. The data are the mean ± s.e.m. The results were analyzed using one-way ANOVA with Tukey’s post-hoc test.

## Data Availability

The datasets used and analyzed in this study are available from the corresponding author upon reasonable request.

## Abbreviations

AKT: Protein kinase B
AMPK: AMP-activated protein kinase
CM: Conditioned media
EGFR: Epidermal growth factor receptor
EMT: Epithelial–mesenchymal transition
GLUT1: Glucose transporter 1
HK2: Hexokinase 2
IL-6: Interleukin-6
IL-6R: IL-6 receptor
intraperitoneal: I.p.
LDHA: Lactate dehydrogenase A
M1: Classically activated macrophages
M2: Alternatively activated macrophages
MCP-1: Monocyte chemoattractant protein-1
MCTs: Monocarboxylate transporters
NK: Natural killer
2-NBDG: (2-Deoxy-2-[(7-nitro-2,1,3-benzoxadiazol-4-yl)amino]-d-glucose)
OXPHOS: Oxidative phosphorylation
PARP: Poly (ADP-ribose) polymerase
PD-1: Programmed death-1
PD-L1: Programmed death-ligand 1
PI: Propidium iodide
PKM2: Pyruvate kinase 2
PMA: Phorbol myristate acetate
qRT–PCR: Quantitative real-time polymerase chain reaction
RPIA: Ribose 5-phosphate isomerase A
TAM: Tumor-associated macrophage
TME: Tumor microenvironment
TNBCs: Triple-negative breast cancers
Tregs: Regulatory T cells
WST-1: (4[3-(4-Iodophenyl)-2(4-nitrophenyl)-2*H*-5-tetrazolio]-1,3-benzene disulfonate)

## References

[1] Collignon J, Lousberg L, Schroeder H, Jerusalem G (2016). Triple-negative breast cancer: treatment challenges and solutions. Breast Cancer (Dove Med Press).

[2] Oliveras-Ferraros C, Vazquez-Martin A, Lopez-Bonet E, Martin-Castillo B, Del Barco S, Brunet J (2008). Growth and molecular interactions of the anti-EGFR antibody cetuximab and the DNA cross-linking agent cisplatin in gefitinib-resistant MDA-MB-468 cells: new prospects in the treatment of triple-negative/basal-like breast cancer. Int J Oncol.

[3] Arun B, Akar U, Gutierrez-Barrera AM, Hortobagyi GN, Ozpolat B (2015). The PARP inhibitor AZD2281 (Olaparib) induces autophagy/mitophagy in BRCA1 and BRCA2 mutant breast cancer cells. Int J Oncol.

[4] Han Y, Li CW, Hsu JM, Hsu JL, Chan LC, Tan X (2019). Metformin reverses PARP inhibitors-induced epithelial–mesenchymal transition and PD-L1 upregulation in triple-negative breast cancer. Am J Cancer Res.

[5] Wang H, Zhang X, Teng L, Legerski RJ (2015). DNA damage checkpoint recovery and cancer development. Exp Cell Res.

[6] Kwa MJ, Adams S (2018). Checkpoint inhibitors in triple-negative breast cancer (TNBC): where to go from here. Cancer.

[7] Li Z, Qiu Y, Lu W, Jiang Y, Wang J (2018). Immunotherapeutic interventions of triple-negative breast cancer. J Transl Med.

[8] Li CW, Lim SO, Chung EM, Kim YS, Park AH, Yao J (2018). Eradication of triple-negative breast cancer bells by targeting glycosylated PD-L1. Cancer Cell.

[9] Zhou J, Tang Z, Gao S, Li C, Feng Y, Zhou X (2020). Tumor-associated macrophages: recent insights and therapies. Front Oncol.

[10] Jayasingam SD, Citartan M, Thang TH, Mat Zin AA, Ang KC, Ch'ng ES (2019). Evaluating the polarization of tumor-associated macrophages into M1 and M2 phenotypes in human cancer tissue: technicalities and challenges in routine clinical practice. Front Oncol.

[11] Sun X, Wang M, Wang M, Yu X, Guo J, Sun T (2020). Metabolic reprogramming in triple-negative breast cancer. Front Oncol.

[12] McKnight SL (2010). On getting there from here. Science.

[13] Hussein YR, Bandyopadhyay S, Semaan A, Ahmed Q, Albashiti B, Jazaerly T (2011). Glut-1 expression correlates with basal-like breast cancer. Transl Oncol.

[14] Oh S, Kim H, Nam K, Shin I (2017). Glut1 promotes cell proliferation, migration and invasion by regulating epidermal growth factor receptor and integrin signaling in triple-negative breast cancer cells. BMB Rep.

[15] Macheda ML, Rogers S, Best JD (2005). Molecular and cellular regulation of glucose transporter (GLUT) proteins in cancer. J Cell Physiol.

[16] Avanzato D, Pupo E, Ducano N, Isella C, Bertalot G, Luise C (2018). High USP6NL levels in breast cancer sustain chronic AKT phosphorylation and GLUT1 stability fueling aerobic glycolysis. Cancer Res.

[17] Lunt SY, Vander Heiden MG (2011). Aerobic glycolysis: meeting the metabolic requirements of cell proliferation. Annu Rev Cell Dev Biol.

[18] Fitton JH, Stringer DN, Karpiniec SS (2015). Therapies from fucoidan: an update. Mar Drugs.

[19] Fitton HJ, Stringer DS, Park AY, Karpiniec SN (2019). Therapies from fucoidan: new developments. Mar Drugs.

[20] Vetvicka V, Vetvickova J (2017). Fucoidans stimulate immune reaction and suppress cancer growth. Anticancer Res.

[21] Hentati F, Tounsi L, Djomdi D, Pierre G, Delattre C, Ursu AV (2020). Bioactive polysaccharides from seaweeds. Molecules.

[22] Lin Y, Qi X, Liu H, Xue K, Xu S, Tian Z (2020). The anti-cancer effects of fucoidan: a review of both in vivo and in vitro investigations. Cancer Cell Int.

[23] Sanjeewa KKA, Jeon YJ (2021). Fucoidans as scientifically and commercially important algal polysaccharides. Mar Drugs.

[24] Jin JO, Chauhan PS, Arukha AP, Chavda V, Dubey A, Yadav D. The therapeutic potential of the anticancer activity of fucoidan: current advances and hurdles. Mar Drugs. 2021;19(5). 10.3390/md19050265 PMC8151601 34068561

[25] Sun J, Sun J, Song B, Zhang L, Shao Q, Liu Y (2016). Fucoidan inhibits CCL22 production through NF-κB pathway in M2 macrophages: a potential therapeutic strategy for cancer. Sci Rep.

[26] Tomori M, Nagamine T, Miyamoto T, Iha M. Evaluation of the immunomodulatory effects of fucoidan derived from *Cladosiphon okamuranus* Tokida in Mice. Mar Drugs. 2019;17(10). 10.3390/md17100547 PMC6835671 31554251

[27] Chen LM, Liu PY, Chen YA, Tseng HY, Shen PC, Hwang PA (2017). Oligo-fucoidan prevents IL-6 and CCL2 production and cooperates with p53 to suppress ATM signaling and tumor progression. Sci Rep.

[28] Chen LM, Tseng HY, Chen YA, Al Haq AT, Hwang PA, Hsu HL (2020). Oligo-fucoidan prevents M2 macrophage differentiation and HCT116 tumor progression. Cancers (Basel).

[29] Wu SY, Chen YT, Tsai GY, Hsu FY, Hwang PA (2020). Protective effect of low-molecular-weight fucoidan on radiation-induced fibrosis through TGF-β1/Smad pathway-mediated inhibition of Collagen I accumulation. Mar Drugs.

[30] Hwang PA, Lin HV, Lin HY, Lo SK (2019). Dietary supplementation with low-molecular-weight fucoidan enhances innate and adaptive immune responses and protects against *Mycoplasma pneumoniae* antigen stimulation. Mar Drugs.

[31] Yan MD, Lin HY, Hwang PA (2019). The anti-tumor activity of brown seaweed oligo-fucoidan via lncRNA expression modulation in HepG2 cells. Cytotechnology.

[32] Shih HJ, Chu KL, Wu MH, Wu PH, Chang WW, Chu JS (2012). The involvement of MCT-1 oncoprotein in inducing mitotic catastrophe and nuclear abnormalities. Cell Cycle.

[33] Gorelik R, Gautreau A (2014). Quantitative and unbiased analysis of directional persistence in cell migration. Nat Protoc.

[34] Weng YS, Tseng HY, Chen YA, Shen PC, Al Haq AT, Chen LM (2019). MCT-1/miR-34a/IL-6/IL-6R signaling axis promotes EMT progression, cancer stemness and M2 macrophage polarization in triple-negative breast cancer. Mol Cancer.

[35] Crowe AR, Yue W (2019). Semi-quantitative determination of protein expression using immunohistochemistry staining and analysis: an integrated protocol. Bio Protoc.

[36] Tung NM, Boughey JC, Pierce LJ, Robson ME, Bedrosian I, Dietz JR (2020). Management of hereditary breast cancer: American Society of Clinical Oncology, American Society for Radiation Oncology, and Society of Surgical Oncology guideline. J Clin Oncol.

[37] Yu Y, Liang Y, Li D, Wang L, Liang Z, Chen Y (2021). Glucose metabolism involved in PD-L1-mediated immune escape in the malignant kidney tumour microenvironment. Cell Death Discov.

[38] Cha JH, Yang WH, Xia W, Wei Y, Chan LC, Lim SO (2018). Metformin promotes antitumor immunity via endoplasmic-reticulum-associated degradation of PD-L1. Mol Cell.

[39] Gutierrez-Salmeron M, Garcia-Martinez JM, Martinez-Useros J, Fernandez-Acenero MJ, Viollet B, Olivier S (2020). Paradoxical activation of AMPK by glucose drives selective EP300 activity in colorectal cancer. PLoS Biol.

[40] Lin SC, Hardie DG (2018). AMPK: sensing glucose as well as cellular energy status. Cell Metab.

[41] Al-Khalili L, Bouzakri K, Glund S, Lonnqvist F, Koistinen HA, Krook A (2006). Signaling specificity of interleukin-6 action on glucose and lipid metabolism in skeletal muscle. Mol Endocrinol.

[42] Marko DM, Foran G, Vlavcheski F, Baron DC, Hayward GC, Baranowski BJ (2020). Interleukin-6 treatment results in GLUT4 translocation and AMPK phosphorylation in neuronal SH-SY5Y cells. Cells.

[43] Colegio OR, Chu NQ, Szabo AL, Chu T, Rhebergen AM, Jairam V (2014). Functional polarization of tumour-associated macrophages by tumour-derived lactic acid. Nature.

[44] de la Cruz-Lopez KG, Castro-Munoz LJ, Reyes-Hernandez DO, Garcia-Carranca A, Manzo-Merino J (2019). Lactate in the regulation of tumor microenvironment and therapeutic approaches. Front Oncol.

[45] Arroyo-Crespo JJ, Arminan A, Charbonnier D, Deladriere C, Palomino-Schatzlein M, Lamas-Domingo R (2019). Characterization of triple-negative breast cancer preclinical models provides functional evidence of metastatic progression. Int J Cancer.

[46] Kaur P, Nagaraja GM, Zheng H, Gizachew D, Galukande M, Krishnan S (2012). A mouse model for triple-negative breast cancer tumor-initiating cells (TNBC-TICs) exhibits similar aggressive phenotype to the human disease. BMC Cancer.

[47] Ossovskaya V, Koo IC, Kaldjian EP, Alvares C, Sherman BM (2010). Upregulation of poly (ADP-ribose) polymerase-1 (PARP1) in triple-negative breast cancer and other primary human tumor types. Genes Cancer.

[48] Zhang C, Yu X, Gao L, Zhao Y, Lai J, Lu D (2017). Noninvasive imaging of CD206-positive M2 macrophages as an early biomarker for post-chemotherapy tumor relapse and lymph node metastasis. Theranostics.

[49] Mittendorf EA, Philips AV, Meric-Bernstam F, Qiao N, Wu Y, Harrington S (2014). PD-L1 expression in triple-negative breast cancer. Cancer Immunol Res.

[50] Planes-Laine G, Rochigneux P, Bertucci F, Chretien AS, Viens P, Sabatier R (2019). PD-1/PD-L1 targeting in breast cancer: the first clinical evidences are emerging. A literature review. Cancers (Basel).

[51] Christofk HR, Vander Heiden MG, Harris MH, Ramanathan A, Gerszten RE, Wei R (2008). The M2 splice isoform of pyruvate kinase is important for cancer metabolism and tumour growth. Nature.

[52] Miao Y, Zhang LF, Guo R, Liang S, Zhang M, Shi S (2016). (18)F-FDG PET/CT for monitoring the response of breast cancer to miR-143-based therapeutics by targeting tumor glycolysis. Mol Ther Nucleic Acids.

[53] Ma C, Zu X, Liu K, Bode AM, Dong Z, Liu Z (2019). Knockdown of pyruvate kinase M inhibits cell growth and migration by reducing NF-kB activity in triple-negative breast cancer cells. Mol Cells.

[54] Zhou Z, Li M, Zhang L, Zhao H, Sahin O, Chen J (2018). Oncogenic kinase-induced PKM2 tyrosine 105 phosphorylation converts nononcogenic PKM2 to a tumor promoter and induces cancer stem-like cells. Cancer Res.

[55] Huang X, Li X, Xie X, Ye F, Chen B, Song C (2016). High expressions of LDHA and AMPK as prognostic biomarkers for breast cancer. Breast.

[56] Morais-Santos F, Granja S, Miranda-Goncalves V, Moreira AH, Queiros S, Vilaca JL (2015). Targeting lactate transport suppresses in vivo breast tumour growth. Oncotarget.

[57] Lim SO, Li CW, Xia W, Lee HH, Chang SS, Shen J (2016). EGFR signaling enhances aerobic glycolysis in triple-negative breast cancer cells to promote tumor growth and immune escape. Cancer Res.

