# Supplementary material for: Oligo-Fucoidan supplementation enhances the effect of Olaparib on preventing metastasis and recurrence of triple-negative breast cancer in mice
Source: J Biomed Sci. 2022 Sep 15;29:70. doi: 10.1186/s12929-022-00855-6 (PMC9479298; doi:10.1186/s12929-022-00855-6)
Supplement: Supplementary file 1 — Additional file 1: Fig. S1. PD-L1 is suppressed by DBPR112, Oligo-Fucoidan and olaparib. PD-L1 levels in HCC1395 cells were examined after treatment with DBPR112 (5 ng/ml), Oligo-Fucoidan (400 μg/ml), olaparib (50 μM) or anti-PD-L1 (50 μM) for 48 h. Fig. S2. Olaparib and Oligo-Fucoidan treatment do not affect CD4(+) T cells, B cells or NK cells in lymphoid system. (A) Representative FACS plots showing the gating strategy for the immunophenotyping of splenic T-, B- and NK-cell subpopulations. Cytotoxic T cells, T helper cells, B cells and NK cells were separated from the lymphocyte gate via CD8(+) vs. side scatter (SSC), CD4(+) vs. SSC, CD19(+) vs. SSC and NK1.1(+) vs. SSC, respectively. Analysis of CD4(+) (B), NK1.1(+) (C) and CD19(+) (D) subpopulations showed that neither monotherapy nor dual treatment with olaparib and Oligo-Fucoidan affected the abundance of CD4(+) T helper cells, B cells, or NK cells. Vehicle (PBS), n=8; olaparib, n=9; Fucoidan, n=9; olaparib and Fucoidan combination, n=7. (E) Representative FACS plots delineating recurrent tumor-infiltrating Tregs. Tregs were identified by CD25(+) and CD127(-) staining of CD4(+) cells. The data are the mean ± s.e.m. The results were analyzed using one-way ANOVA with Tukey’s post-hoc test. [file 12929_2022_855_MOESM1_ESM.docx]

**Oligo-Fucoidan supplementation enhances the effect of Olaparib on preventing metastasis and recurrence of triple-negative breast cancer in mice**

Li-Mei Chen^1§^, Pao-Pao Yang^1§^, Aushia Tanzih Al Haq^1§^, Pai‐An Hwang^2^, You-Chen Lai^1^, Yueh-Shan Weng^1^, Michelle Audrey Chen^1^, Hsin-Ling Hsu^1^*

**Supplementary Information**

**Supplementary Fig. 1.** PD-L1 is suppressed by DBPR112, Oligo-Fucoidan and olaparib. PD-L1 levels in HCC1395 cells were examined after treatment with DBPR112 (5 ng/ml), Oligo-Fucoidan (400 μg/ml), olaparib (50 μM) or anti-PD-L1 (50 μM) for 48 h.

**
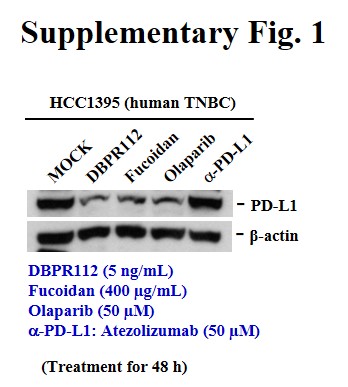
**

**Supplementary Fig. 2.** Olaparib and Oligo-Fucoidan treatment do not affect CD4(+) T cells, B cells or NK cells in lymphoid system. **(A)** Representative FACS plots showing the gating strategy for the immunophenotyping of splenic T-, B- and NK-cell subpopulations. Cytotoxic T cells, T helper cells, B cells and NK cells were separated from the lymphocyte gate via CD8(+) vs. side scatter (SSC), CD4(+) vs. SSC, CD19(+) vs. SSC and NK1.1(+) vs. SSC, respectively. Analysis of CD4(+) **(B)**, NK1.1(+) **(C)** and CD19(+) **(D)** subpopulations showed that neither monotherapy nor dual treatment with olaparib and Oligo-Fucoidan affected the abundance of CD4(+) T helper cells, B cells, or NK cells. Vehicle (PBS), n=8; olaparib, n=9; Fucoidan, n=9; olaparib and Fucoidan combination, n=7. **(E)** Representative FACS plots delineating recurrent tumor-infiltrating Tregs. Tregs were identified by CD25(+) and CD127(-) staining of CD4(+) cells. The data are the mean ± s.e.m. The results were analyzed using one-way ANOVA with Tukey’s post-hoc test.

**
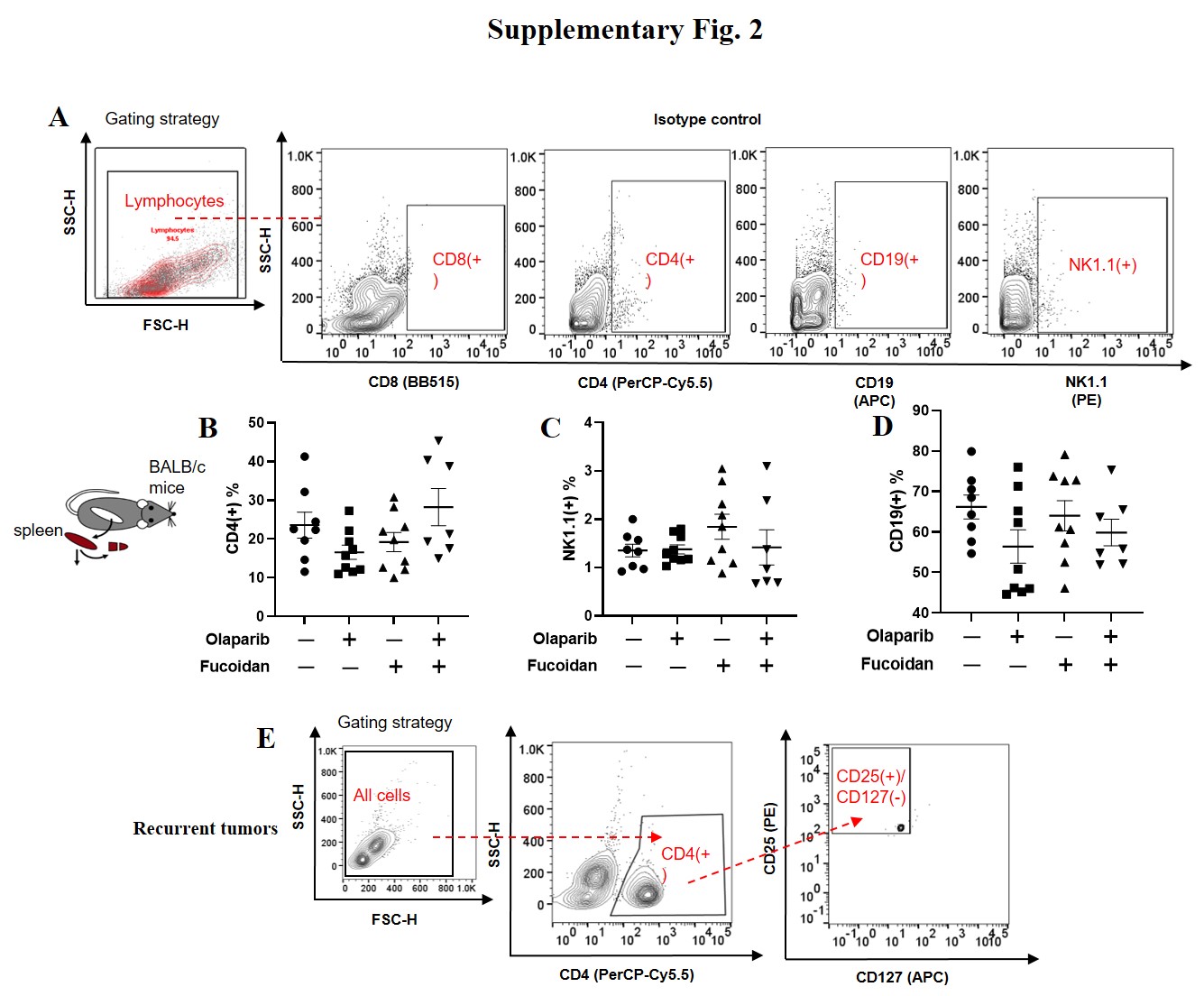
**
